# A School Health Project Can Uplift the Health Status of School Children in Nepal

**DOI:** 10.1371/journal.pone.0166001

**Published:** 2016-11-03

**Authors:** Rachana Manandhar Shrestha, Moe Miyaguchi, Akira Shibanuma, Arun Khanal, Junko Yasuoka, Masamine Jimba

**Affiliations:** 1 Department of Community and Global Health, Graduate School of Medicine, The University of Tokyo, 7-3-1, Hongo, Bunkyo-ku, Tokyo, 113–0033, Japan; 2 Tritunga Development Society (Tritunga Bikas Samaj), P. O. Box 8162, New Baneshwor, Kathmandu, Nepal; Leibniz Institute for Prvention Research and Epidemiology BIPS, GERMANY

## Abstract

**Background:**

School health is effective in helping students achieve health literacy, enhance their health-related behaviors, and thereby improve their health status. However, in resource-limited countries, evidence is limited to show the impact of school health. We determined the association of the school health and nutrition (SHN) project activities on students’ a) health knowledge, b) hygiene practices, and c) health outcomes, one year after the project completion.

**Methods:**

This is a cross-sectional study conducted among the schools with the SHN project and without the project in four districts of Nepal. We recruited 604 students from six schools in the project group and 648 students from other six schools in the comparison group. We used a self-administered questionnaire to collect the data, and analyzed them using regression models and a structural equation model (SEM).

**Results:**

Students from the SHN project group reported the decreased odds of worm infestation (AOR = 0.50, 95% CI: 0.34 to 0.75) and diarrhea/ dysentery infection (AOR = 0.67, 95% CI: 0.47 to 0.97) compared to those in the comparison group. Furthermore, the SEM analysis also showed that the students in the project group were more likely to have better health outcomes *(β = 0*.*03*, *p< 0*.*05)*.

**Conclusion:**

Students in the SHN project group were more likely to have better health outcomes compared to those in the comparison group, even after one year of the project completion. As it can bring about sustainable changes for students, it should be scaled up in other parts of the country.

## Introduction

Schools are in a unique position to promote health of school children [1–3]. According to the World Health Organization (WHO), school health is an effective intervention that can simultaneously improve the quality of education and health. It has a potential to help students achieve health literacy, enhance their health-related behaviors, and thereby improve their health status [4–7].

Over the years, school health has advanced from class-room based health education to a comprehensive and integrated approach. It has been focusing on school health policies, life skills-based health education, health services, and a supportive school environment for health promotion [8, 9]. In 1995, WHO launched a Global School Health Initiative to advocate for improved school health [10] by increasing the quantity and quality of ‘Health-Promoting Schools’ HPS) [10, 11]. In addition to WHO, several other United Nations (UN) agencies united together to develop Focusing Resources on Effective School Health (FRESH) framework, an overarching school health model [9]. Since then, thousands of schools throughout the world introduced better school health programs to promote health of school children [8, 9].

In Nepal, around 40% of the total population is 16 years or younger [12], which is the group of school-age children. However, little attention has been paid on their health issues [13]. It neither falls under the priority of health workers nor under the school management team [14]. In the past, some donor-initiated school health projects [15] were run, but only sporadically for several years [13, 16].

In the last decade, however, the Government of Nepal has recognized the need to ensure better health and improved learning of school children. In 2006, the Ministry of Health and Population (MOHP) and the Ministry of Education and Sports (MOES) of Nepal jointly endorsed the National School Health and Nutrition (SHN) Strategy [14]. Since the strategy was endorsed, the two ministries have been working together with the relevant stakeholders from the national to community level to put the strategy into practice [14]. The strategy is comprehensive and has four objectives [14, 15]. They are 1) to improve the use of SHN services by school children, 2) to improve school environment, 3) to improve health and nutrition behaviors and habits and, 4) to improve and strengthen community support systems and policy environment [14].

Based on the strategy, MOHP, MOES, and the Japan International Cooperation Agency (JICA) jointly launched a school health and nutrition (SHN) project in two districts from 2008 to 2012. The project included activities such as annual physical check-up, deworming, iron supplementation, first aid services, special health education, child club mobilization, provision of safe drinking water and toilets, mid-day meal, etc. It also focused on improving students’ health knowledge, hygiene practices, and nutritional behaviors [13, 15].

A growing body of literature has shown that school health can promote health of students, school personnel, families and other members of the community [1, 9, 17], but the evidence is limited particularly in resource-limited countries. Nepal is not an exception. Only a handful of studies are available focusing on particular health issues of school students [18–20]. Furthermore, few studies have evaluated school health projects and their effects on students' health outcomes in Nepal. In this study, we determined the association of the SHN project activities on students’ a) health knowledge, b) hygiene practices, and c) health outcomes, one year after the project completion.

## Methods

### Study design and area

In this cross-sectional study, we collected data from two groups of students from four districts in Nepal in 2013, one year after the SHN project completion. The first group was the project group from the schools in Sindhupalchok and Syangja districts, which had participated in the SHN project [14, 15]. The second group was the comparison group from Dolakha and Tanahu districts. We selected the second group for comparison because the SHN project had covered all the schools in the target districts, and Dolakha and Tanahu are also the neighboring districts with similar socio demographic backgrounds.

### Study participants and sampling

The SHN project was implemented in all the schools in the target districts. A total of 546 schools in Syangja and 567 schools in Sindhupalchok participated in the project. We purposively selected three schools, each from two project districts, from which 604 students participated in the study. The district education office recommended these schools as average, above average, and below average schools based on their performance on SHN activities during the project. The students from this group were exposed to various SHN project activities such as regular students' health check-up, iron and Vitamin A supplementation, mass deworming, provision of school tiffin or mid-day meal, maintaining students' health record, provision of safe and adequate drinking water, provision of separate and adequate toilets, maintenance of first aid kits, promotion of tin-box library/IEC corners and teachers' training for conducting SHN activities [21]. Similarly, we recruited 648 students from six schools from two comparison districts, three from each. The schools in this group were not part of the SHN project and were devoid of many SHN activities. The students were only exposed to basic health education curriculum, basic hygiene and sanitation facilities, and deworming program by the government.

The students belonged to grades six, seven and eight. We randomly selected one class from each grade. Every class consisted of 30 to 35 students, resulting in approximately 100 students from each school. We included all the students (n = 1,252) who were present on the day of data collection and agreed to participate in the study. The response rate was 100%. However, based on the national data of the average attendance rate in the four districts [22–24], we assumed that 10 to 25% of the total enrolled students might not have been included in this study.

We used Power and Precision software, version 4 (Biostat, Englewood, NJ, USA) to calculate the sample size. The minimum sample size was calculated as 326 for each group of students for 80% power, with the level of significance set at 5% for a 95% confidence interval and with 90% response rate. We considered a 10% difference in response rates. We then adjusted it by assuming within-school intra-class correlation coefficient of 0.01 for health outcomes [25]. Then, we calculated the design effect as 1.29, using the formula D_eff_ = 1+(m-1)* p, where m is the number of students in each class and p is inter-class correlation. Thus, the required number of students in each group was estimated to be around 420, which was obtained by multiplying minimum sample size with the design effect. To account for missing data, we estimated that the sample size should be about 600 in each group.

### Instrument development

We developed the questionnaire in English by adopting and modifying questions from the following five survey questionnaires: (1) the Global School-based Health Survey (GSHS) questionnaire [26], (2) the Health Behavior in School-aged Children (HBSC) survey questionnaire [27], (3) ‘wash in schools: monitoring package’ [28], (4) student questionnaire from the SHN project survey, and (5) questions from a previous study conducted in Hong Kong [4]. We then translated the questionnaire into Nepali, back translated into English, and verified the original and back-translated versions. After that, we pre-tested the questionnaire among 100 students before the data collection of the main survey. Finally, the first author discussed its contents among public health researchers, school health experts, schoolteachers, and students. Based on the pre-test results and feedbacks, we modified the questionnaire.

### Measures and instruments

#### Socio-demographic variables

The socio-demographic variables measured in this study included age, gender, grade, ethnicity, religion, living arrangement, parents’ education level, and parents’ occupation.

#### SHN activities

**a) School health services.** We measured the health services available in the schools through the variables extracted from the strategic objectives of the National SHN strategy [14]. They were mass deworming, vitamin A and iron supplementation, vision, hearing and dental screenings, students’ health records, and first aid services. We asked the students if they had received the above-mentioned health services in their schools in the last one year. Students responded to the items as ‘1 = yes’, ‘2 = no’ and ‘3 = don’t know’. We assumed that students who responded ‘don’t know’ to the items were not aware about the services and may not have used the services in their schools. We then recoded the responses into two categories by grouping ‘no’ and ‘don’t know’ as 0 and ‘yes’ as 1. Furthermore, we calculated the total score of the school health services. The score ranged from zero to eight. A higher score indicated better excess to school health services available in the schools.

b) Health and sanitation facilities

We asked the students if their schools had the facilities such as safe drinking water, toilet and hand washing facilities. Students’ responses were categorized as ‘1 = yes’, ‘2 = no’ and ‘3 = don’t know’. We assumed that the students who responded ‘don’t know’ to the items have not seen and may not have used those facilities in their schools. We then recoded their responses into two categories by grouping ‘no’ and ‘don’t know’ as 0 and ‘yes’ as 1. Moreover, we calculated the total score ranging from zero to eight, higher score indicating better access to health and sanitation facilities.

c) Child club and special health classes

We also asked students if their schools had child clubs for SHN activities and special health classes providing life skill-based education. We extracted related-variables from the strategic objectives of the National SHN strategy [14]. Students responded to the items as ‘1 = yes’, ‘2 = no’ and ‘3 = don’t know’. We then recoded the responses into two categories by grouping ‘no’ and ‘don’t know’ as 0 and ‘yes’ as 1.

#### Health knowledge

We measured health-related knowledge using nine items. We asked students about the health knowledge and information they received from their schools. Students' responses were categorized as ‘1 = yes’, ‘2 = no’ and ‘3 = don’t know’. We then recoded the responses into two categories by grouping ‘no’ and ‘don’t know’ as 0 versus ‘yes’ as 1. Furthermore, we calculated a total score of the nine items ranging from 0 to 9. A higher score indicated more health-related knowledge or information received from schools and vice versa.

#### Hygiene practices

We used four items to measure the students' hygiene practices which included hand washing, brushing teeth, and sanitary practices. For brushing teeth, students responded to the item ‘During the past 30 days, how many times did you brush your teeth per day?’ The responses ranged from ‘1 = never’ to ‘4 = 2 or more times per day’. The responses were recoded into two categories as ‘0 = one or less than one time per day’ and ‘1 = two or more times per day’ [29]. Moreover, students responded to the items on hand hygiene practices such as ‘During the past 30 days, how often did you wash your hands before eating?’ ‘…after using the toilet or latrine?’ and ‘…how often did you use soap when washing your hands?’ The responses were in an ordinal scale ranging from 1(never) to 4 (always), which we recoded into two categories as ‘0 = never to many times’ and ‘1 = always’ [29]. We then calculated the total score ranging from 0 to 4, higher score indicating better hygiene practices.

#### Health outcomes

We assessed students’ oral health status, prevalence of diarrhea or dysentery, and worm infestation during the past one month to measure health outcomes. Students responded to the item ‘During the past 12 months how often did you have a toothache or feel discomfort because of your teeth?’. The responses were in an ordinal scale ranging from 1(never) to 4 (always). We then recoded them as ‘0 = sometimes to always’ and ‘1 = never’. Furthermore, students responded to the items such as ‘Did you suffer from diarrhea or dysentery within past one month?’ ‘….worm infestation within past one month?’ The responses were categorized as ‘1 = yes’, ‘2 = no’ and ‘3 = don’t know’, which we later recoded into two categories by grouping ‘yes’ as 0 and ‘no’ and ‘don’t as 1. We assumed that those students who responded ‘don’t know’ to the items had not experienced pain or discomfort and became ill because of those health conditions. Furthermore, we calculated the total score ranging from 0 to 3, higher score indicating better health outcomes.

### Data collection

We collected the data in November and December 2013 and trained local research assistants on the data collection and ethical procedures before the data collection. Students filled out self-administered questionnaire in Nepali-language during their regular class hours, which took 40–50 minutes to complete. The first author provided instructions to the students before the data collection. The research assistants were present throughout the process to answer students’ queries.

### Data analysis

Out of 1,252 recruited students, data sets of five students were incomplete and were not included in the analysis. We then analyzed 1,247 data sets, 603 from the project group and 644 from the comparison group. We conducted chi-squared test to check the independence of the socio-demographic characteristics, variables related to school health services, health and sanitation facilities, child clubs, special health classes, hygiene practices and health outcomes between the two groups of students. We also conducted the independent sample t-test to examine the difference in knowledge scores.

We conducted multiple logistic regression analyses to examine the differences in the variables of school health services, health and sanitation facilities, child clubs and special health classes, by adjusting potential confounders. We also conducted a multivariable linear regression analysis to determine the difference in knowledge score between students from the project group and the comparison group. Furthermore, we conducted logistic regression analyses to examine the significance difference in hygiene practices and health outcomes between the two groups of students. The variables included in the models did not have multicollinearity. Multiple regression analysis did not permit simultaneous analyses of all the variables in this study and only one dependent variable could be tested at a time [30, 31]. Finally, we used structural equation model (SEM) and assessed direct and indirect relationships between all the independent and dependent variables concurrently to show the association of the project activities on students' health knowledge, hygiene practices and health outcomes [31].

We used SPSS version 16.0 for Windows (SPSSInc., Chicago, IL) and Stata 12.1 software (StataCorp LP, College Station, TX, USA) for all statistical analyses. The level of significance was set at p< 0.05 for all the statistical analyses.

### Ethical consideration

The ethical application and consent procedure of this study were reviewed and approved by the Research Ethics Committee of the University of Tokyo and the Nepal Health Research Council (NHRC). The district education offices also permitted data collection from the schools in all four districts. We distributed letters to all the schools requesting for their cooperation and participation. The school principals provided written consents for their students’ participation. Furthermore, we distributed letters to the parents/ guardians of the targeted students to explain our study in advance and requested students to obtain their verbal consent, which was not recorded. Students, who received the consent from their parents/ guardians, were explained about the details of this study and then they signed the informed consent forms. They were also ensured for their voluntary participation and they could withdraw from the study at any time. We managed the data with high confidentiality and kept the participants’ identity anonymous.

## Results

### Socio demographic characteristics of the students

Table 1 shows the general characteristics of the students from both groups. Of the 1,247 participants, 603 (48.4%) students were from the project group and 644 (51.6%) were from the comparison group. The mean age of students in the project group was 12.3 years (SD 1.3) and 13.5 years (SD 1.4) in the comparison group. Hindu was the major religion in both groups. The majority of students from both groups belonged to Janajati ethnic group. Also, about 70% of the students in both groups were living with both of their parents. Regarding their parents' education level, about 60% of fathers had completed schooling up to lower secondary level in both groups. Sixty to seventy percent of the mothers had also studied up to lower secondary level in both groups.

**Table 1:** Socio demographic characteristics of the students (N = 1,247).

	Schools with the SHN project (n = 603)		Schools without the SHN project (n = 644)		
Variable	Mean	SD	Mean	SD	p-value
**Age** ‡	12.8	1.3	13.5	1.4	**<0.001**
	**N**	**%**	**N**	**%**	
**Gender** **†**					
Male	257	42.6	282	43.8	0.677
Female	346	57.4	362	56.2	
**Grade** **†**					
Grade 6	161	26.7	175	27.1	0.911
Grade 7	213	35.3	220	34.2	
Grade 8	229	38.0	249	38.7	
**Ethnicity** **†**					
Brahmin/Chhetri	240	39.9	193	30.0	**<0.001**
Janajati	283	47.0	298	46.2	
Dalit	79	13.1	153	23.8	
**Religion** **†**					
Hindu	410	68.1	549	85.5	**<0.001**
Buddhist	181	30.1	65	10.1	
Other	11	1.8	28	4.4	
**Living arrangement** **†**					
Both parents	397	65.8	459	71.2	**0.043**
One parent	34	5.6	41	6.4	
Others	172	28.6	144	22.4	
**Father’s education** **†**					
Illiterate	44	7.3	74	11.6	**0.019**
Up to lower secondary	349	58.0	370	58.1	
Secondary and above	209	34.7	193	30.3	
**Mother’s education** **†**					
Illiterate	92	15.3	167	26.6	**<0.001**
Up to lower secondary	416	69.3	401	63.9	
Secondary and above	92	15.4	60	9.5	

†, Chi-square test

‡, T-test

### SHN activities in the schools

Table 2 presents the similarities and differences in the school health services, health and sanitation facilities, child clubs and provision of special health education classes in both groups. Students reported significantly higher proportion of school health services in the project group. Most of the following school health services were more accessible in schools of the project group: deworming (89.8% vs. 54.6%, p<0.001), vitamin A supplementation (37.4% vs. 18.0%, p<0.001), iron supplementation (26.5% vs. 13.1%, p<0.001), first aid services (91.4% vs. 79.4%, p<0.001), vision screening (51.4% vs. 39.2%, p<0.001), hearing screening (20.1% vs. 6.9%, p<0.001) and maintenance of school health record (61.3% vs. 44.6%, p<0.001).

**Table 2:** SHN activities in the schools (N = 1,247).

	Schools with the SHN project		Schools without the SHN project		
Variables	n	%	n	%	p-value
**School health services**					
Deworming †					
Yes	539	89.8	347	54.6	**<0.001**
No/ Don’t know	61	10.2	289	45.4	
Vitamin A †					
Yes	223	37.4	115	18.0	**<0.001**
No/ Don’t know	374	62.6	523	82.0	
Iron tablets †					
Yes	158	26.5	83	13.1	**<0.001**
No/ Don’t know	439	73.5	553	86.9	
First aid services †					
Yes	543	91.4	508	79.4	**<0.001**
No/ Don’t know	51	8.6	132	20.6	
Vision screening †					
Yes	308	51.4	250	39.2	**<0.001**
No/ Don’t know	291	48.6	388	60.8	
Hearing screening †					
Yes	120	20.1	44	6.9	**<0.001**
No/ Don’t know	477	79.9	594	93.1	
Dental screening †					
Yes	94	15.7	110	17.2	0.495
No/ Don’t know	503	84.3	530	82.8	
Students' health records †					
Yes	366	61.3	287	44.6	**<0.001**
No/ Don’t know	231	38.7	356	55.4	
**Health and sanitation facilities**					
Enough water for drinking †					
Yes	512	85.4	573	89.1	**0.046**
No/ Don’t know	88	14.6	56	10.9	
Presence of a toilet †					
Yes	594	98.8	630	98.1	0.312
No/ Don’t know	7	1.2	12	1.9	
Separate toilets for boys and girls †					
Yes	593	98.8	623	97.5	0.081
No/ Don’t know	7	1.2	16	2.5	
Water available for toilets †					
Yes	534	88.9	586	91.1	0.179
No/ Don’t know	67	11.1	57	8.9	
Place to wash hands after toilet use †					
Yes	561	93.7	542	84.2	**<0.001**
No/ Don’t know	38	6.3	102	15.8	
Place to wash hands before eating †					
Yes	524	87.0	550	86.2	0.727
No/ Don’t know	78	13.0	88	13.8	
Enough water to wash hands †					
Yes	541	90.5	603	93.6	**0.039**
No/ Don’t know	57	9.5	41	6.4	
Soap to wash hands †					
Yes	300	50.0	241	37.7	**<0.001**
No/ Don’t know	300	50.0	399	62.3	
**Child club for SHN activities** †	476	79.5	367	57.3	**<0.001**
Yes					
No/ Don’t know	123	20.5	274	42.7	
**Special health classes** †					
Yes	439	73.0	445	69.1	0.125
No/ Don’t know	162	27.0	199	30.9	

†, Chi-square test

Two of eight items measuring health and sanitation facilities were significantly higher in the project group: place to wash hand after toilet use (93.7% vs. 84.2%, p<0.001) and soap to wash hand (50.0% vs. 37.7%, p<0.001). Moreover, the following facilities were highly accessible in both groups: toilet (98.8% vs. 98.1%), separate toilets for boys and girls (98.8% vs. 97.5%), and place to wash hands before eating (87.0% vs. 86.2%).

According to the students, child club activities (79.5% vs. 57.3%, p<0.001) were significantly higher in the project group. Though statistically insignificant, more students in the project group reported that they had special health classes in their schools (73.0% vs. 69.1%).

### Students’ health knowledge, hygiene practices, and health outcomes

Table 3 shows the minimal difference in the mean knowledge scores of the students from the project group (7.4, SD 2.1) and the comparison group (7.8, SD 1.7). The former group reported a slightly higher proportion of hygiene practices such as hand washing before eating (54.9% vs. 50.8%), hand washing after toilet use (77.4% vs. 76.4%), using soap while hand washing (49.9% vs. 47.5%), and brushing teeth twice or more times per day (59.0% vs. 54.3%). However, the results were not statistically significant. Furthermore, the prevalence of diarrhea/dysentery (18.9% vs. 23.7%, p = 0.038) and worm infestation (14.4% vs. 22.1%, p = 0.001) was significantly lower among the students in the project group.

**Table 3:** Students' health knowledge, hygiene practices and health outcomes (N = 1,247).

Variable	Schools with the SHN project				Schools without the SHN project				
	N	Mean		SD	N	Mean		SD	p-value
Health knowledge ‡	581	7.40		2.1	612	7.80		1.7	<0.001
		N	%		N		%	p-value	
**Hygiene practices**									
During the past 30days, how often did you wash your hands before eating? †									
Never to many times		272	45.1		317		49.2	0.146	
Always		331	54.9		327		50.8		
During the past 30days, how often did you wash your hands after using the toilet or latrines? †									
Never to many times		136	22.6		152		23.6	0.661	
Always		467	77.4		492		76.4		
During the past 30 days, how often did you use soap when washing your hands? †									
Never to many times		302	50.1		338		52.8	0.396	
Always		301	49.9		306		47.5		
How often do you brush your teeth? †									
**≤** One time per day		247	41.0		293		45.7	0.096	
≥ Two times per day		355	59.0		348		54.3		
**Health outcomes**									
How often did you have a toothache because of your teeth? †									
Sometimes to always		291	48.6		305		47.7	0.745	
Never		308	51.4		335		52.3		
Did you suffer from diarrhea or dysentery within past one month? †									
Yes		113	18.9		151		23.7	**0.038**	
No/ Don’t know		485	81.1		485		76.3		
Did you suffer from worm infestation within past one month? †									
Yes		86	14.4		140		22.1	**0.001**	
No/ Don’t know		501	85.6		493		77.9		

†, Chi-square test

‡, T-test

### Comparison of the SHN activities

Table 4 depicts the results of multiple logistic regression models for school health services, health and sanitation facilities, child clubs and special health classes. After adjusting for covariates and confounders, the schools in the project group had increased odds of school health services: deworming (AOR = 7.35, 95% CI: 5.28 to 10.24), vitamin A supplementation (AOR = 2.70, 95% CI: 2.04 to 3.59), iron tablet supplementation (AOR = 2.20, 95% CI: 1.60 to 3.03), first aid services (AOR = 3.04, 95% CI: 2.09 to 4.43), vision screening (AOR = 1.71, 95% CI: 1.35 to 2.20), hearing screening (AOR = 3.61, 95% CI: 2.39 to 5.43) and the maintenance of students’ school health records (AOR = 2.17, 95% CI: 1.69 to 2.80). Similarly, the odds of the following hand washing facilities were also significantly higher in the project group: place to wash hands after toilet use (AOR = 2.51, 95% CI: 1.65 to 3.80), and soap to wash hands (AOR = 1.60, 95% CI: 1.25 to 2.05). The presence of child club for the SHN activities was also significantly higher (AOR = 2.93, 95% CI: 2.23 to 3.85) in the project group.

**Table 4:** Comparison of the SHN activities in the schools.

Variable	AOR	95% CI for AOR
**School health services in school**		
Deworming tablets †	7.35***	(5.28−10.24)
Vitamin A †	2.70***	(2.04−3.59)
Iron tablets †	2.20***	(1.60−3.03)
First aid services †	3.04***	(2.09−4.43)
Vision screening †	1.71***	(1.35−2.20)
Hearing screening †	3.61***	(2.39−5.43)
Dental screening †	0.92	(0.66−1.28)
Students’ health records †	2.17***	(1.69−2.80)
**Health and sanitation facilities**		
Enough water for drinking †	0.83	(0.57−1.19)
Presence of toilets †	1.64	(0.59−4.58)
Separate toilets for boys and girls †	2.43	(0.93−6.34)
Water for toilets †	0.76	(0.51−1.14)
Place to wash hands after toilet use †	2.51***	(1.65−3.80)
Place for wash hands before eating †	1.22	(0.85−1.74)
Enough water for washing hands †	0.68	(0.43−1.07)
Soap to wash hands †	1.60***	(1.25−2.05)
**Child club for SHN activities** **†**	2.93***	(2.23−3.85)
**Special health classes** **†**	1.16	(0.89−1.51)

*, p<0.05

**, p<0.01

***,p<0.001

†, Adjusted for age, gender, ethnicity, religion, living arrangement, father’s education, and mother’s education

### Comparison of students' health knowledge score, hygiene practices and health outcomes

In Table 5, students from the project group reported decreased odds of worm infestation (AOR = 0.50, 95% CI: 0.34 to 0.75) as well as diarrhea/ dysentery infection (AOR = 0.67, 95% CI: 0.47 to 0.97), after controlling for covariates and confounders. This group of students also showed increased odds of hand washing practice before eating (AOR = 1.32, 95% CI: 1.01 to 1.73). However, their health knowledge score was relatively lower compared to students in the comparison group (β = -0.55, 95% CI: -0.90 to -0.19).

**Table 5:** Comparison of students' health knowledge score, hygiene practices and health outcomes.

Variable	Beta (adjusted)	95% CI
**Health knowledge** †	-0.55**	(-0.90 −-0.19)
	**AOR**	**95% CI**
**Hygiene practices**		
Wash your hands before eating ‡	1.32*	(1.01−1.73)
Wash your hands after using the toilet ‡	1.06	(0.77−1.47)
Use soap when washing your hands ‡	1.21	(0.92−1.58)
Brush your teeth ‡	1.16	(0.89−1.51)
**Health outcomes**		
Toothache ††	0.84	(0.63−1.12)
Diarrhea or dysentery within past one month ††	0.67*	(0.47−0.97)
Worm infestation within past one month ††	0.50**	(0.34−0.75)

*, p<0.05

**, p<0.01

***,p<0.001

†, Adjusted for age, gender, ethnicity, religion, living arrangement, father’s education, mother’s education, child club for SHN activities and special health classes

‡, Adjusted for age, gender, ethnicity, religion, living arrangement, father’s education, mother’s education, health and sanitation facilities score, child club, special health classes and health knowledge score

††, Adjusted for age, gender, ethnicity, religion, living arrangement, father’s education, and mother’s education, school health services score, health and sanitation facilities score, child club, special health classes, health knowledge score and hygiene practices score

### SEM showing direct, indirect and total effect of the SHN project

**Fig 1:**
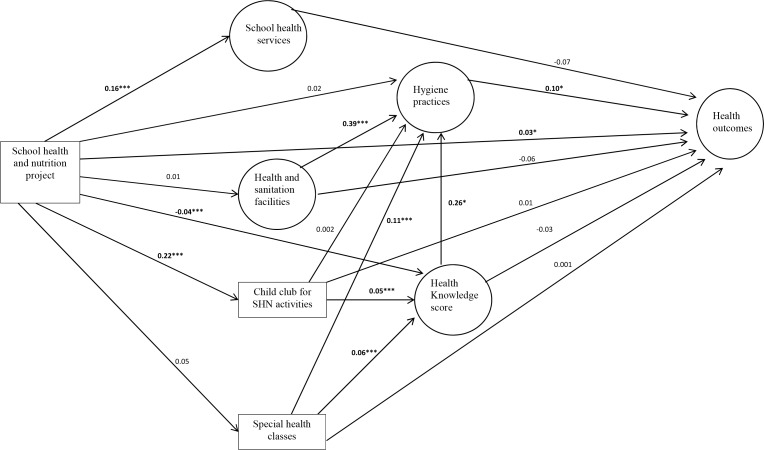
SEM showing direct and indirect paths between SHN project activities, students' health knowledge, hygiene practices, and health outcomes. Model Fit: Chi-square = 4391.866, RMSEA = 0.056. * p<0.05; ** p<0.01; *** p<0.001.

In Table 6 and Fig 1, we have presented the results of SEM with standardized coefficients and the model showing direct and indirect paths. The model showed the evidence of adequate fit with root mean square error of approximation (RMSEA) = 0.056 [32, 33], and chi-square = 4391.9. The total effects from SEM analysis in Table 6 showed that the students in the project group were more likely to have better health outcomes (β = 0.03, p< 0.05). However, students in this group were less likely to have higher health knowledge (β = -0.03, P< 0.01).

**Table 6:** SEM showing direct, indirect and total effect of the SHN project.

	Direct effect			Indirect effect			Total effect		
	Coef.	95%CI		Coef.	95%CI		Coef.	95%CI	
Variable		Lower	Upper		Lower	Upper		Lower	Upper
**School health services**	0.16***	0.11	0.20				0.16***	0.11	0.20
**Health and sanitation facilities**	0.01	-0.01	0.03				0.01	-0.01	0.03
**Child club for SHN activities**	0.22***	0.17	0.28				0.22***	0.17	0.28
**Special health classes**	0.05	-0.01	0.11				0.05	-0.01	0.11
**Health knowledge**	-0.04***	-0.06	-0.03	0.01***	0.01	0.02	-0.03**	-0.05	-0.01
**Hygiene practices**	0.02	-0.02	0.07	0.002	-0.01	0.01	0.03	-0.02	0.07
**Health outcomes**	0.03*	0.01	0.06	-0.01*	-0.01	-0.01	0.03*	0.01	0.05

*, p<0.05

**, p<0.01

***,p<0.001

The direct effects from SEM analysis in showed that the schools in the project group were also more likely to have better access to school health services (β = 0.16, P< 0.001) and had more child club activities (β = 0.22, P< 0.001). Furthermore in Fig 1, presence of more child club activities (β = 0.05, P< 0.001) and more special health classes (β = 0.06, P< 0.001) in schools were more likely to increase students’ health knowledge score. Though the students in the project group had lower health knowledge score (β = -0.04, P< 0.01), higher health knowledge score was associated with better hygiene practices (β = 0.26, P< 0.05). Better access to health and sanitation facilities (β = 0.34, P< 0.001) and having more special health classes (β = 0.11, P< 0.001) in school were also associated with better hygiene practices. Finally, better hygiene practices were more likely to improve students' health outcomes (β = 0.10, P< 0.05).

The indirect path in Fig 1 illustrated that the SHN project was indirectly associated with better health outcomes via the mediators: child club activities, health knowledge score and hygiene practices. The indirect association was also seen between the SHN project and better health outcomes via the mediators: health knowledge score and hygiene practices.

## Discussion

This is the first study in Nepal, which has determined the association of the SHN project activities on students' health knowledge, hygiene practices and health outcomes, one year after the project completion. Moreover, this study is the one among a few school health intervention studies, which has applied SEM to analyze and understand complex relationships between multiple variables simultaneously. The SEM analysis showed that students in the project group were more likely to have better health outcomes, even one year after the project completion.

In this study, a significantly higher proportion of students in the project group had better access to various school health services than those in the comparison group. Of all the health services, deworming service given to students was seven times higher. According to the students in this group, their schools had significantly better access to hand washing soap and place to wash hands after toilet use, and conducted more child club activities than in the comparison schools. Though the project sustainability is one of the major hurdles within school setting [34], the schools in the project group could still continue some SHN activities for more than a year, after the project completion.

Furthermore, the prevalence of worm infestation and diarrhea/ dysentery infection were significantly lower in the project group, even one year after the project ended. The endline survey also reported a decreased prevalence of diarrhea and worm infestation [21]. Deworming might have played a major role in lowering the gastrointestinal infections [35, 36]. The above results imply that the students' positive health outcomes in the project group may be attributed to better SHN activities.

In this study, SEM analysis has shown direct and indirect relationships between all the dependent and independent variables concurrently. The analysis showed that the schools in the project group were more likely to have better access to school health services and had more child club activities. Moreover, students in this group were more likely to have better health outcomes. The SEM analysis further showed that presence of more child club activities and special health classes in schools were associated with students’ higher health knowledge score. Also, having more special health classes in the school and students' higher health knowledge was associated with better hygiene practices. The above findings suggest that having more child club activities and special health classes might have contributed to better hygiene practices by improving students’ health knowledge. This could be because child club activities included school cleaning, organizing health related activities, operating library, management of first aid kit, and others [21]. Mobilizing child clubs for SHN activities was one of the major activities of the SHN project. A previous study has also shown that children had opportunities to gain knowledge and learn skills for their personal development through child clubs [37].

The SEM analysis further showed that better access to health and sanitation facilities in schools were associated with better hygiene practices among students. Therefore in the project group, better access to hand washing facilities might have encouraged students to use soap and wash their hands before eating and after toilet use. Finally, SEM showed that better hygiene practices among students were more likely to improve their health outcomes. Our results are comparable to those of previous studies, which reported similar association between personal hygiene practices and health education interventions among the students [38, 39], which could reduce gastrointestinal illnesses [40–42].

In this study, the difference in the mean health knowledge scores was minimal between the two groups. Although the difference was statistically significant, it is of little practical importance. This minimal difference could be found because the students from both groups had been exposed to basic health education and hygiene issues as part of compulsory health education curriculum of Nepal [41].

### Limitations

The results should be interpreted with three study limitations. First, this is a cross sectional study, therefore causality cannot be established. However, the study results showed that the SHN project activities had positive association with students' health outcomes and school health facilities. Second, we did not have baseline data to compare the change over a period of time within the same group of students. To overcome this limitation, we included the students’ data from the schools without the SHN project from the neighboring districts. Third, the students self-reported the questionnaire, which might have led to over or under-reporting, leading to social-desirability bias. However, we collected the data in absence of schoolteachers and kept student identities anonymous.

## Conclusion

The schools in the project group had significantly better access to school health services, health and sanitation facilities, and had more child club activities. Students in this group were more likely to have better hygiene practices and health outcomes compared to those in the comparison group. These results are encouraging and indicate the potential of the SHN project to improve students’ health outcomes, even one year after the project completion. Thus, the project activities should be scaled up in other parts of the country. Also, longitudinal studies should be conducted on school health projects in Nepal to confirm the causality.
